# MRI Versus Histopathology: Evaluating Diagnostic Accuracy in Detecting Endometrioma

**DOI:** 10.7759/cureus.88713

**Published:** 2025-07-25

**Authors:** Aneeqa Qureshi, Junaid Iqbal, Saman Shah, Saba Akram, Mallick Muhammad Zohaib Uddin, Anam Hafeez, Anam Naz, Uffan Zafar

**Affiliations:** 1 Radiology, Dr. Ziauddin Hospital, Karachi, PAK; 2 Radiology, Aga Khan University Hospital, Karachi, PAK; 3 Obstetrics and Gynecology, Jinnah Postgraduate Medical Center, Karachi, PAK; 4 Pathology/Histopathology, Aga Khan University Hospital, Karachi, PAK; 5 Radiology, Patel Hospital, Karachi, PAK; 6 Radiology, University of Texas Medical Branch at Galveston, Galveston, USA

**Keywords:** diagnostic accuracy, endometrioma, endometriosis, gynecology, histopathology, imaging, mri, radiology, women imaging

## Abstract

Introduction

Endometriosis is a condition characterized by the presence of ectopic endometrial glands surrounded by endometrial stroma. When this occurs within the ovaries, it is referred to as an endometrioma. It commonly affects women of reproductive age and typically presents as cyclical lower abdominal pain. Diagnosing endometriosis remains a clinical challenge. Magnetic resonance imaging (MRI) is considered more specific than sonography for detecting endometriomas.

Objective

This cross-sectional study aimed to assess the diagnostic accuracy of MRI in detecting endometriomas, using histopathological findings as the gold standard.

Subject and methods

A total of 139 women suspected of having endometriomas based on clinical symptoms were included in the study. Data were collected using a structured, interview-based questionnaire, which covered sociodemographic information, clinical symptoms, sonographic findings, MRI results, and histopathological confirmation.

Results

The mean age of participants was 25.68 ± 4.49 years. MRI showed a sensitivity of 95.9% (93/97), specificity of 85.7% (36/42), positive predictive value (PPV) of 93.9% (93/99), negative predictive value (NPV) of 80.0% (36/40), and an overall diagnostic accuracy of 92.8%.

Conclusion

The findings indicate that MRI has high sensitivity, specificity, and overall accuracy in detecting endometriomas, supporting its role as a reliable diagnostic tool.

## Introduction

Endometriosis is a pathology defined by the presence of endometrial glands, endometrial stroma, and hemosiderin-laden macrophages outside the uterine corpus at ectopic locations. It is a common ailment in women of the second and third decades and initially manifests as cyclic abdominal and pelvic pain. In the later stages, it causes infertility and is a contributor in 15% of women with infertility [1]. The most commonly involved sites are the posterior portion of the broad ligament and the ovaries [2]. The other involved sites include the gastrointestinal tract, omentum, urinary tract, and soft tissue, and rarely the lungs too. When it is present within the ovaries, it is labelled as an endometrioma, endometriotic cyst, or a chocolate cyst. These endometriotic foci bleed and then, in reaction, are walled off by the adhesions or become buried within the tissues, resulting in the formation of endometrioma [3].

In most cases, the diagnosis of endometriosis is challenging for clinicians, researchers, and patients as it presents with variable symptoms. Ovarian endometriomas are even more tricky to diagnose due to the overlapping presentations with other adnexal masses and ailments, such as luteal or simple hemorrhagic cysts. The management and outcome of these entities are different. For example, for luteal cysts and simple hemorrhagic cysts, surgical resection is usually indicated, whereas endometriomas have been associated with poor pregnancy outcomes [4] and are refractory to medical management [5].

Ultrasonography has been used as a diagnostic tool for endometrioma. However, evidence-based literature about transabdominal sonography shows different sonographic patterns for endometrioma and lacks efficiency in the specific characterization of endometriomas [4-7]. However, transvaginal sonography (TVS) has better diagnostic accuracy for endometriomas as reported by Leone et al. [8] in their study on the spectrum of TVS findings in 38 histologically proven endometriomas. In their study, 82% of the patients with endometriomas had a characteristic sonographic pattern of a "cystic pelvic mass with homogeneous hypo-echoic low-level echoes." Other studies also showed that TVS has a sensitivity of 82%-84%, with a specificity of 90% in detecting endometriomas, with similar patterns [9].

However, in the modern-day world, such a large margin of error is simply not acceptable to the patients. This is where the need for a better, more accurate diagnostic procedure is required. Magnetic resonance imaging (MRI) is one such diagnostic procedure. It becomes a modality of choice for indeterminate findings of sonography in ovarian masses. The specific radiological findings of endometriomas in MRI are high signal intensity at both T1- and T2-weighted sequences, which persist at subsequent fat-suppressed T1-weighted images [10]. This fat suppression helps in differentiating endometriotic cysts from other cystic lesions like teratomas, playing a mandatory role in establishing the diagnosis of endometrioma [11]. Chronic bleeding and accumulation of iron and proteins in the endometriotic cavity are visualized as shading in MRI, which is a gradual change in intensity of signals at T2-weighted images [12,13].

This specific feature helps in differentiating the functional hemorrhagic cyst from endometrioma; the hemorrhagic cysts do not show any shading and disappear at follow-up imaging. The use of diagnostic criteria like T1 hyper-intense cysts with T2 shading or multiple T1 hyper-intense cysts irrespective of T2 signal intensity in MRI shows the sensitivity and specificity of as high as 90% and 98%, respectively, and an accuracy of 96% in diagnosing endometriomas [12]. However, in women with biopsy-proven endometriomas, the sensitivity and specificity of MRI for detecting endometrioma were 69% and 75%, respectively [14].

MRI is thus more specific than sonography in the diagnosis of endometrioma and can be used as an imaging examination without any risk of exposure to harmful radiation, like other imaging modalities (such as computed tomography). Our study uses the diagnostic accuracy of MRI as a primary diagnostic imaging modality to test in our study setting and explores the positive and negative predictive value of MRI in diagnosing endometrioma. This research is particularly important because there is no existing local literature on the subject, and our research shall fill this large gap in the literature, providing valuable data that may be used for future in-depth research.

## Materials and methods

Study design and setting

This cross-sectional study was conducted at the Department of Radiology, Ziauddin University Hospital, Karachi, over a period of six months from January 2020 to June 2020. A total of 139 female patients suspected of having endometrioma were included using a non-probability, consecutive sampling method.

Eligibility criteria

Inclusion Criteria

Married female patients aged 18-45 years who presented with suspected endometrioma were included in the study. Suspicion was based on clinical symptoms (such as pelvic pain and subfertility), physical examination findings (e.g., adnexal mass or nodularity, tenderness), and sonographic features, including cysts with diffuse low-level internal echoes, multilocularity, hyperechoic wall foci, abnormal linear thickening, or nodules or masses in specific subperitoneal sites.

Exclusion Criteria

Patients were excluded if they were in the menstruation phase at the time of imaging, were using oral contraceptive pills or other hormonal therapies, were pregnant, or were postmenopausal.

Data collection procedure

After obtaining ethical approval from the competent authority and informed consent from all patients, data were collected using a structured interview-based questionnaire. The questionnaire included inquiries about sociodemographic details, clinical signs and symptoms, sonographic findings, MRI impressions, and the confirmatory diagnosis based on histopathology. A senior radiologist with a minimum of three years of experience interpreted the MRI and prepared the final report. Histopathological evaluations were performed by a consultant histopathologist, also with at least three years of experience, following standard procedures for biopsy sample extraction, slicing (into 1 cm sections), staining with hematoxylin and eosin (H&E), and microscopic examination at appropriate magnifications. Patient confidentiality was maintained by assigning coded identifiers instead of names, and all data were stored in a password-protected system. The data were discarded upon completion of the project.

Data analysis procedure

Data analysis was performed using Microsoft Excel 2016 (Microsoft Corp., Redmond, WA, USA) and IBM SPSS Statistics for Windows, Version 21.0 (Released 2012; IBM Corp., Armonk, NY, USA). Qualitative variables, including marital status, types of presenting symptoms and complaints, and positive findings on sonography, MRI, and histopathology, were presented as frequencies and percentages. Quantitative variables such as age, age at menarche, duration of symptoms, and number of abortions or miscarriages were summarized using means and standard deviations. Diagnostic performance metrics, sensitivity, specificity, positive predictive value (PPV), negative predictive value (NPV), and accuracy, were calculated.

Effect modifiers such as age, marital status, clinical signs and symptoms, duration of symptoms, and age at menarche were controlled through stratification. Diagnostic accuracy was then assessed post-stratification.

## Results

**Figure 1:**
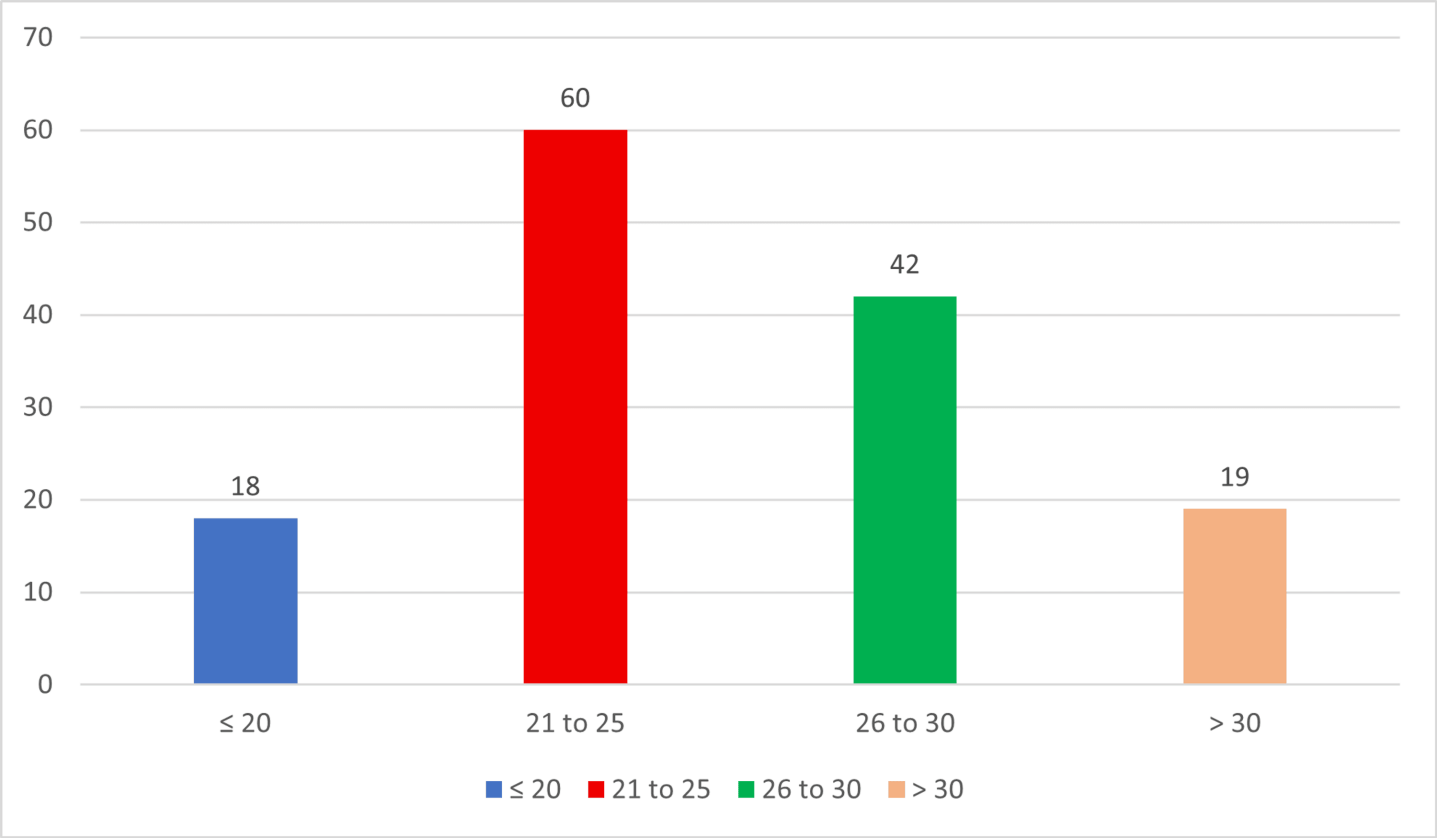
Age distribution of the patients (n = 139)

A total of 139 women with suspected endometrioma, based on clinical presentation such as lower abdominal pain, infertility, adnexal masses, and tenderness on physical examination, were included in the study. The participants had a mean age of 25.68 ± 4.49 years, with the age distribution illustrated in Figure 1. Demographic variables, including age at menarche and duration of symptoms, are presented in Table 1. The mean age at menarche was 12.90 ± 1.34 years, and the average duration of symptoms was 6.02 ± 1.42 weeks. Among patients with a history of miscarriage, the average number of abortions per participant was one.

**Table 1:** Demographic variables of the women included in the study

Variables	Mean	Std. deviation	
			Age (years)	25.68	4.49	
Age of menarche (years)	12.9	1.342	
Duration of symptoms (weeks)	6.02	1.422	
Number of abortions (count)	1	-	

The most commonly reported presenting complaint was pelvic pain, affecting 109 (78.4%) of the women, followed by ovarian masses or nodules and tenderness, each present in 67 (48.2%) cases. Other symptoms and complaints, such as subfertility, were also reported, as shown in Table 2.

**Table 2:** Presenting symptoms and complaints

Presenting symptoms and complaints	Frequency	Percentage
Pelvic pain	109	78.4%
Subfertility	49	35.3%
Adnexal mass/nodules	67	48.2%
Tenderness	67	48.2%
Cysts	42	30.2%

**Figure 2:**
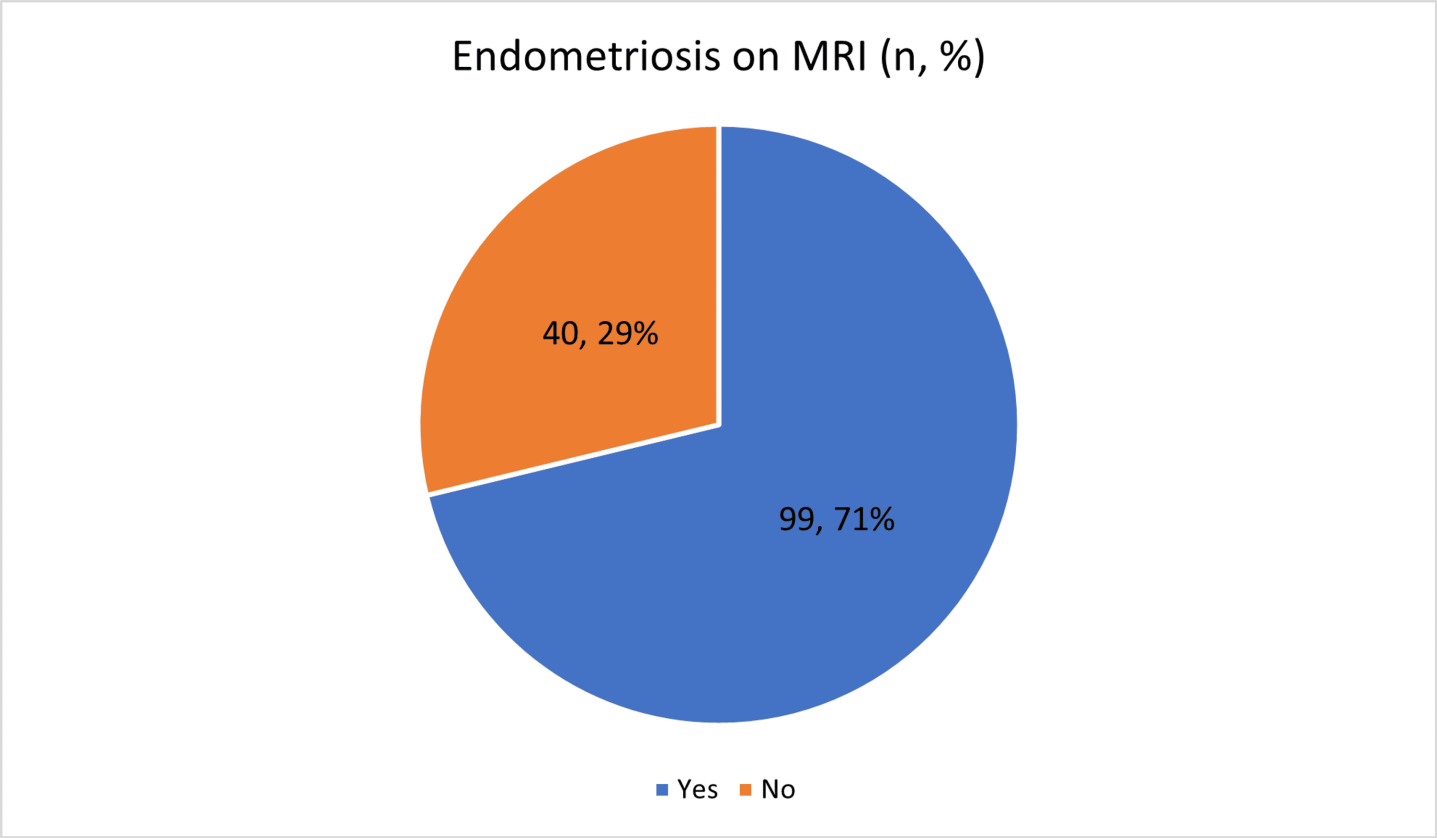
MRI findings in endometrioma detection

**Figure 3:**
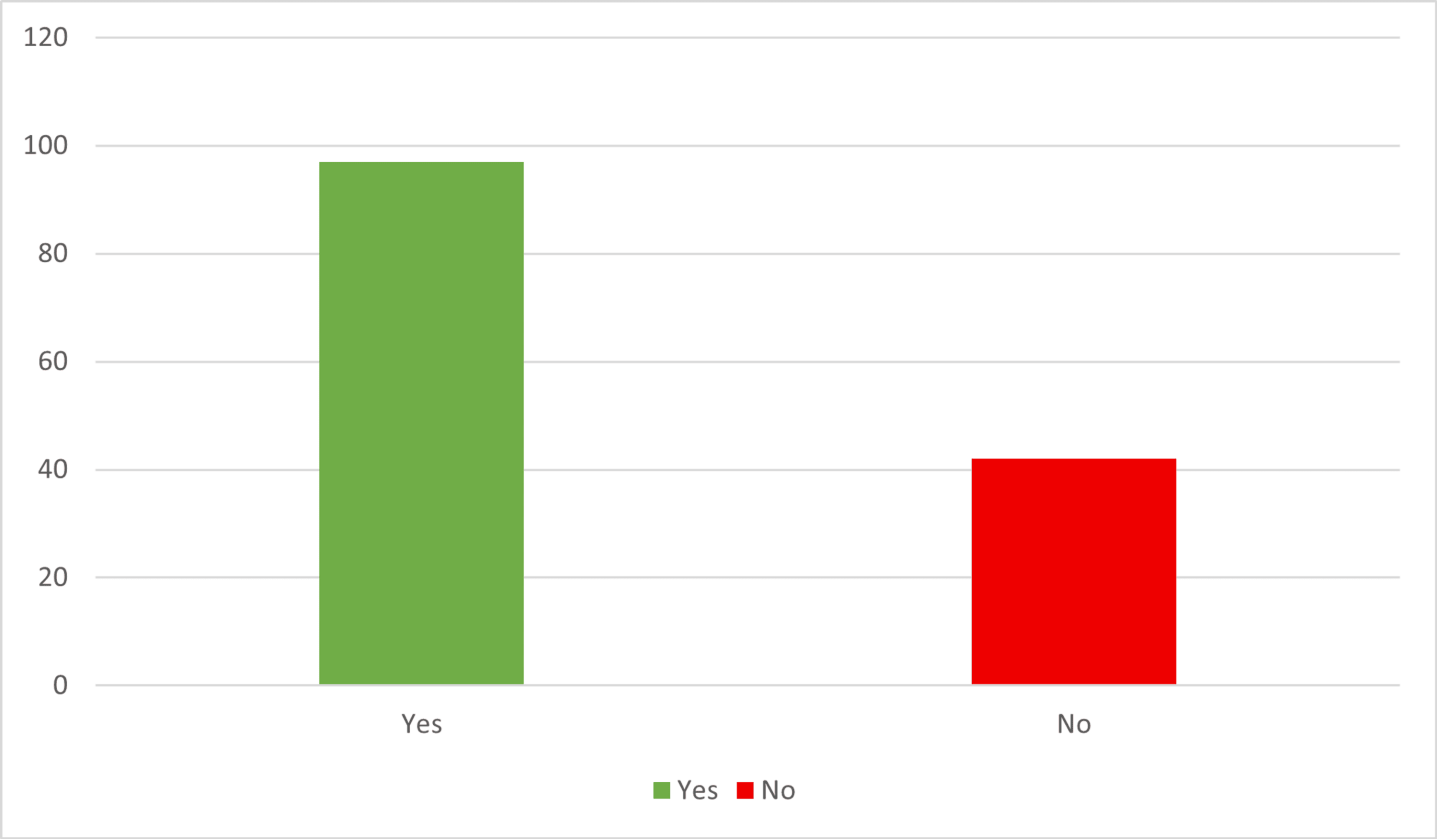
Histopathological confirmation of endometrioma (n = 139)

MRI established a diagnosis of endometrioma in 99 out of 139 cases, representing 71.22% of the study population (Figure 2). Histological examination of biopsy samples confirmed endometrioma in 69.78% of cases (Figure 3).

A comparison of MRI and biopsy findings enabled a detailed analysis of MRI’s diagnostic accuracy. The study summarized key parameters, including sensitivity, specificity, PPV, and NPV, with the results presented in Table 3 and Table 4.

**Table 3:** MRI findings for endometrioma detection evaluated against histopathology, which served as the gold standard

MRI findings	Histopathology positive	Histopathology negative	Total n (%)
Positive	93	6	99 (71.2%)
Negative	4	36	40 (28.8%)
Total n (%)	97 (69.8%)	42 (30.2%)	139

**Table 4:** Diagnostic accuracy parameters of MRI in detecting endometrioma PPV: positive predictive value, NPV: negative predictive value.

Parameter	Value
Sensitivity	95.9%
Specificity	85.7%
PPV	93.9%
NPV	80%
Accuracy	92.8%

The sensitivity of MRI for diagnosing endometrioma was 95.9% (93/97), indicating a high probability of detecting the condition when it is present. The specificity was 85.7% (36/42), suggesting that MRI is also effective in ruling out endometrioma when it is absent. The PPV was 93.9% (93/99), meaning that when MRI indicated endometrioma, histopathology confirmed the diagnosis in nearly 94% of cases. Similarly, the NPV was 80.0% (36/40), showing that in cases where MRI did not detect endometrioma, histopathology also confirmed its absence in 80% of cases. These results yield an overall diagnostic accuracy of 92.8%, highlighting MRI as a highly reliable tool for diagnosing endometrioma.

Stratification of the data by demographic variables, including age, age at menarche, and duration of symptoms, demonstrated consistently high diagnostic accuracy across subgroups (Table 5). MRI sensitivity was 95.2% in patients with symptom duration of six weeks or less, compared to 97.1% in those with symptoms lasting more than six weeks. Among women with an age at menarche of ≤ 13 years, sensitivity was 93.8%, while it was 100% in those with menarche after age 13. When stratified by age, sensitivity was 86.8% in women aged ≤ 25 years and 94.3% in those older than 25 years. These findings further support the diagnostic reliability of MRI across various patient demographics, underscoring its value in detecting endometrioma.

**Table 5:** Diagnostic accuracy of MRI in detecting endometrioma, taking histopathology as the gold standard, stratified by age, age at menarche, and duration of symptoms PPV: positive predictive value, NPV: negative predictive value.

	Duration of symptoms less than or equal to six weeks	Duration of symptoms more than or equal to six weeks	Age of menarche ≤ 13 years	Age of menarche > 13 years	Age ≤ 25 years	Age > 25 years
Sensitivity	95.2%	97.1%	93.8%	100%	86.8%	94.3%
Specificity	88.6%	71.4%	92%	76.5%	81.3%	88.5%
PPV	93.7%	94.4%	96.8%	89.2%	85.2%	91.7%
NVP	91.2%	83.3%	85.2%	100%	86.7%	92%
Accuracy	92.7%	92.8%	93.2%	92%	93.6%	91.8%

The diagnostic accuracy of MRI was also evaluated in relation to specific clinical presenting symptoms, including chronic pelvic pain, subfertility, tenderness, adnexal masses or nodules, and cysts, with results summarized in Table 6. MRI demonstrated a sensitivity of 96.9% in patients presenting with subfertility, 93.2% in those with tenderness, and 93.7% in cases with adnexal masses or nodules. Specificity was highest (92.6%) in patients with chronic pelvic pain, indicating that MRI was particularly effective in ruling out endometrioma in this group. Additionally, the PPV for chronic pelvic pain was 97.5%, highlighting the strong predictive accuracy of MRI when this symptom is present. However, specificity was notably low (0%) among patients who presented with cysts, suggesting a limitation of MRI in distinguishing endometriomas from other cystic ovarian lesions.

**Table 6:** Diagnostic accuracy of MRI in detecting endometrioma, taking histopathology as the gold standard, stratified by presenting symptoms (chronic pelvic pain, subfertility, tenderness, adnexal masses/nodules, and cysts) PPV: positive predictive value, NPV: negative predictive value.

	Chronic pelvic pain	Subfertility	Tenderness	Adnexal masses/nodules	Cysts
Sensitivity	86.3%	96.9%	93.2%	93.7%	90.5%
Specificity	92.6%	82.4%	87.5%	75%	0%
PPV	97.5%	91.2%	98.2%	98.3%	100%
NVP	89.3%	93.3%	63.6%	42.9%	0%
Accuracy	95.4%	91.8%	92.5%	92.7%	90.4%

The findings of this study demonstrate that MRI is a sensitive, specific, and accurate imaging modality for diagnosing endometrioma, especially in cases with complex clinical presentations or inconclusive sonographic findings. Stratified data analyses further highlight MRI’s reliability and diagnostic efficiency across various patient demographics, reinforcing its value as a dependable tool in the assessment of endometrioma.

## Discussion

The average age of the women in our study was 25.68 ± 4.49 years. In comparison, Alborzi et al. [13] reported a higher mean age of 31 years, while a study by Anwar et al. [14] showed a broader age range of 14 to 84 years, with a mean of 40 ± 13.69 years. These variations in age profiles across studies highlight the widespread nature of endometriosis and underscore the importance of reliable diagnostic tools that perform well across different age groups.

In our study, MRI demonstrated a sensitivity of 95.9% and a specificity of 85.7% in detecting endometrioma. The PPV and PPV were 93.9% and 80%, respectively. These findings align with previously reported results [15,16], supporting the reliability of MRI as a diagnostic modality.

Bazot et al. [17] found that transvaginal sonography (TVS) had a lower sensitivity (78.3%) compared to MRI (84.4%) for detecting uterosacral ligament endometriosis. Notably, more than one-third of their patients, who presented with dysmenorrhea and deep dyspareunia, had negative physical examinations. These findings suggest that MRI should be considered for all symptomatic patients, even when physical examination and TVS are inconclusive. However, further studies are needed to assess the cost-effectiveness of routine MRI use in such cases. The consistently high diagnostic accuracy of MRI, as seen in our study and in the literature, supports its role as a first-line or complementary imaging tool for evaluating endometrioma, particularly in patients with atypical symptoms or inconclusive ultrasound results. In line with this, Lopes et al. [18] proposed a “tenderness-guided” approach to enhance the diagnostic value of TVS, particularly for identifying endometriosis, which may complement the utility of MRI in clinical practice. 

The high diagnostic accuracy observed in our study, along with supporting evidence from previous research, suggests that MRI is a reliable imaging modality for detecting endometrioma. Nevertheless, some limitations must be acknowledged. Our study was conducted at a single center with a relatively small sample size, which may limit the generalizability of the findings. Additionally, MRI interpretation was performed by a single radiologist, and inter-observer variability was not assessed, introducing a potential for interpretational bias. Diagnostic outcomes may vary across institutions depending on the expertise of radiologists and histopathologists. Therefore, future prospective multicenter studies with larger sample sizes and evaluation of inter-observer agreement are recommended to further validate the role of MRI in the diagnosis of endometrioma and to guide optimal patient management.

## Conclusions

In the present study, MRI demonstrated high sensitivity in detecting endometrioma. It is a highly accurate, sensitive, and specific imaging modality that can be routinely used without the risk of exposure to harmful radiation, unlike other modalities such as computed tomography. MRI also offers greater diagnostic value in evaluating large solid and cystic pelvic lesions, complex adnexal masses, and large subserosal pedunculated fibroids that may mimic ovarian malignancy.

## References

[1] Akhtar R, Taj N, Mehnaz S, Furqan A, Khakwani M, Masood H (2017). Subfertile women; frequency of factors leading to tubal blockage evaluated by laparoscopy. Professional Med J.

[2] Chowdhury TS, Mahmud N, Chowdhury TA (2017). Endometriosis: correlation of severity of pain with stages of disease. J Bangladesh Coll Phys Surg.

[3] Shetty SK, Shetty H, Rai S (2016). Laparoscopic evaluation of tubal factor in cases of infertility. Int J Reprod Contracept Obstet Gynecol.

[4] Fatima K, Khanani S (2017). Scar endometriosis: an entity not to be forgotten. J Pak Med Assoc.

[5] Exacoustos C, Zupi E, Piccione E (2017). Ultrasound imaging for ovarian and deep infiltrating endometriosis. Semin Reprod Med.

[6] Guerriero S, Ajossa S, Orozco R, Perniciano M, Jurado M, Melis GB, Alcazar JL (2016). Accuracy of transvaginal ultrasound for diagnosis of deep endometriosis in the rectosigmoid: systematic review and meta-analysis. Ultrasound Obstet Gynecol.

[7] Noventa M, Saccardi C, Litta P (2015). Ultrasound techniques in the diagnosis of deep pelvic endometriosis: algorithm based on a systematic review and meta-analysis. Fertil Steril.

[8] Leone F, Clauser R, Personeni C, Mazzocco M (2016). Ultrasound‐based clinical history of ovarian endometrioma treated by medical therapy: a 10‐year follow‐up study. Ultrasound Obstet Gynecol.

[9] Fraser MA, Agarwal S, Chen I, Singh SS (2015). Routine vs. expert-guided transvaginal ultrasound in the diagnosis of endometriosis: a retrospective review. Abdom Imaging.

[10] Mankar DV, Jain GK (2015). Histopathological profile of ovarian tumours: a twelve year institutional experience. Muller J Med Sci Res.

[11] Ashraf A, Shaikh S, Ishfaq A, Akram A, Kamal F, Ahmad N (2012). The relative frequency and histopathological pattern of ovarian masses. Biomedica.

[12] Kinkel K, Frei KA, Balleyguier C, Chapron C (2006). Diagnosis of endometriosis with imaging: a review. Eur Radiol.

[13] Alborzi S, Rasekhi A, Shomali Z, Madadi G, Alborzi M, Kazemi M, Hosseini Nohandani A (2018). Diagnostic accuracy of magnetic resonance imaging, transvaginal, and transrectal ultrasonography in deep infiltrating endometriosis. Medicine (Baltimore).

[14] Anwar S, Rehan B, Hameed G (2014). MRI for the diagnosis of ultrasonographically indeterminate pelvic masses. J Pak Med Assoc.

[15] Adusumilli S, Hussain HK, Caoili EM (2006). MRI of sonographically indeterminate adnexal masses. AJR Am J Roentgenol.

[16] Togashi K, Nishimura K, Kimura I (1991). Endometrial cysts: diagnosis with MR imaging. Radiology.

[17] Bazot M, Lafont C, Rouzier R, Roseau G, Thomassin-Naggara I, Daraï E (2009). Diagnostic accuracy of physical examination, transvaginal sonography, rectal endoscopic sonography, and magnetic resonance imaging to diagnose deep infiltrating endometriosis. Fertil Steril.

[18] Lopes L, Hindman N, Huang K (2015). Accuracy of magnetic resonance imaging (MRI) in the diagnosis of endometriosis - evaluation of an institutional protocol. J Minim Invasive Gynecol.

